# Surgery for epiretinal membrane using three different surgical platforms: A comparative pilot study

**DOI:** 10.22336/rjo.2025.10

**Published:** 2025

**Authors:** Vibha Badrinath, Ashish Markan, Mohit Dogra, Basavraj Tigari, Deeksha Katoch, Ramandeep Singh

**Affiliations:** 1 Advanced Eye Centre, Postgraduate Institute of Medical Education and Research, Chandigarh, India; 2 Vibha Badrinath and Ashish Markan are combined first authors.

**Keywords:** 3D-HUD, MIOCT, ERM, microscope integrated OCT, standard operating microscope, surgical outcomes, BCVA = Best-corrected visual acuity, FFA = Fundus fluorescein angiography, GAT = Goldman applanation tonometry, IOP = Intraocular pressure, Mi-OCT = Microscope integrated-OCT, OCT = Optical Coherence Tomography, PPV = Pars plana vitrectomy, RPE = Retinal pigment epithelium, SOM = Standard operating microscope, 3D-HUD = 3D head-up display

## Abstract

**Purpose:**

To compare dye-assisted epiretinal membrane (ERM) peeling using a standard operating microscope (SOM) with peeling without staining using either microscope-integrated optical coherence tomography (Mi-OCT) or a three-dimensional heads-up display (3D-HUD) platform.

**Materials and methods:**

A prospective, randomized, and interventional pilot study. Patients requiring surgical intervention for ERM were randomized into group A (Mi-OCT), group B (3D-HUD), where dye was not used, and group C (dye-assisted peeling using SOM). Primary outcomes included the percentage of the eyes where complete ERM removal was possible without staining in groups A and B, intraoperative and postoperative complications, and best-corrected visual acuity (BCVA) at 3 months follow-up. Secondary outcomes included total surgical and ERM peel time.

**Results:**

Complete ERM peeling was possible only in 80% (group A) and 70% (group B) without using dye. Postoperatively, no ERM recurrence was observed in groups A and B, except for one (10%) in group C. BCVA at 3 months improved significantly from baseline in all. The mean surgical and ERM peel time was considerably lesser in groups A and B than in group C.

**Discussion:**

Mi-OCT and 3D-HUD ensured complete ERM removal in 80% and 70% of cases, respectively, without dye, compared to 100% in the conventional group, with one recurrence. Both technologies reduced surgical and peeling time. Intraoperative OCT improved visualization and minimized unnecessary maneuvers, aligning with PIONEER and DISCOVER studies. BCVA improved significantly at three months across all groups, with no intergroup differences. Despite benefits, high costs and a learning curve limit widespread adoption. Our study’s small sample size and short follow-up warrant further research to validate findings and assess long-term outcomes, including potential dye-related toxicity in conventional techniques.

**Conclusion:**

Mi-OCT and 3D-HUD were associated with shorter surgical times and less need for staining. However, they had no added advantage over SOM at the three-month follow-up.

## Introduction

Epiretinal membranes (ERMs) are fibro-cellular semi-transparent membranes formed on the retina’s inner surface. They are formed by the proliferation of retinal glial cells, retinal pigment epithelium (RPE) cells, and hyalocytes on the retinal surface [1]. Most ERMs (cellophane maculopathy) are usually asymptomatic and detected incidentally. They can slowly distort underlying retinal layers (crinkled maculopathy and macular puckers) and produce symptoms like blurred vision, metamorphopsia, micropsia, and monocular diplopia [2,3]. The presence of these visual symptoms warrants surgical intervention. The surgical management of choice is pars plana vitrectomy (PPV) and membrane peeling. Since the advent of chromovitrectomy, most surgeons prefer dye-assisted ERM peeling using a standard operating microscope (SOM). The dye helps to visualize the membrane better, thus assisting in its peeling. With recent reports concerning safety issues with these dyes and their potential damage to retinal photoreceptors, peeling without staining has gained popularity [4].

Recent technological advancements in the field of visualizing systems, like the introduction of microscope integrated-OCT (Mi-OCT) RESCAN 700 (Carl Zeiss Meditec, Germany) and 3D head-up display (3D-HUD) visualizing system integrated with digital filters, have allowed surgeons to visualize these epiretinal membranes without the help of dyes [4-6]. Several studies have separately compared surgical outcomes between SOM and 3D-HUD and SOM and Mi-OCT. Still, no study compares the surgical outcomes with SOM versus Mi-OCT assisted surgery versus 3D HUD surgical platform for ERM peeling. In this pilot study, we aimed to compare dye-assisted peeling using SOM versus peeling without staining using either Mi-OCT or digital filters available with 3D-HUD.

## Materials and methods

This was a prospective, randomized, and interventional pilot study. Patients with either idiopathic or secondary epiretinal membranes were recruited from our center’s retina clinic. The study was conducted under the tenets of the Helsinki Declaration. Ethical approval was obtained from the Postgraduate Institute of Medical Education and Research institutional review board, Chandigarh, India.

The study included patients aged > 18 years with idiopathic or secondary ERMs requiring surgical intervention. It also excluded patients with significant media opacity that did not allow good-quality OCT scans and patients with visual loss due to other causes, such as macular scars, macular degeneration, glaucoma, optic atrophy, etc. Secondary ERMS due to diabetic retinopathy was also excluded.

Patients who met the inclusion criteria were randomized into three groups of 10. Group A included patients who underwent ERM peeling using Mi-OCT without dye staining, group B included patients who underwent 3D-HUD-assisted surgery for ERM removal without dye staining, and group C included patients who underwent conventional dye-assisted ERM peeling using SOM.

A detailed history was taken for all recruited patients. Baseline clinical examination included best-corrected visual acuity (BCVA), intraocular pressure (IOP) using Goldman applanation tonometry (GAT), anterior segment examination, and slit-lamp biomicroscopy using 90D. Peripheral fundus examination was done using the indirect ophthalmoscope with the + 20D lens for all patients at baseline. Color fundus photographs (Visupac FF 450 plus, Carl Zeiss Meditech AG, Germany) were taken to document the posterior segment findings at presentation. Optical coherence tomography (OCT) images were acquired using swept-source OCT DRI OCT Triton (Topcon Corporation, Tokyo, Japan). A 5-line raster scan was done to examine the extent of ERM and underlying retinal thickening and distortion caused by ERM. ERMs were classified as stage 1 to 4, depending upon the presence of ectopic inner foveal layers described by Govetto et al. [7]. Fundus fluorescein angiography (FFA) (Visupac FF 450 plus) was reserved for cases with secondary ERMs to look at the cause (vascular occlusions, vasculitis, etc.), macular perfusion, and assess the visual prognosis.

### Surgical technique

A 25-gauge pars plana vitrectomy (PPV) was done using the Constellation vision system (Alcon Laboratories, Inc., Fort Worth, TX) by a single surgeon (RS). After the core vitrectomy, triamcinolone-assisted posterior vitreous detachment was induced wherever necessary. The completion of a peripheral vitrectomy followed. A +60D Resight lens focused on the posterior pole for ERM peeling.

In group A, Mi-OCT (RESCAN 700 prototype (Carl Zeiss Meditec, Inc., Oberkochen, Germany) was used to perform the surgery. ERM was identified using an OCT scan without using dye to stain the ERM. ERM was peeled off using 25G+ GRIESHABER ILM forceps (Alcon Laboratories, Inc., Fort Worth, TX) by looking at the real-time OCT images generated by Mi-OCT. Mi-OCT ensured complete removal of ERM from the posterior pole. In group B, no dye was used, and the ERM was identified and peeled using 3D HUD with a yellow filter. In group C, ERM peeling was performed on SOM using the conventional technique. Brilliant Blue-green dye (BBG) (Ocublue, Aurolab, Madurai, India) was injected for 1 minute, and excess dye was removed using active suction. ERM was identified with negative staining and peeled off using end-gripping forceps. BBG dye-assisted removal was also done in group A and B eyes, where ERM was not visualized adequately.

Complete ERM peeling was defined as the ability to remove visible ERM in the macular area. No notable attempt was made to peel the ILM in the three groups. Topical antibiotics, steroids, and cycloplegics were given postoperatively.

Patients were followed up on day 1, day 7, one month, and three months postoperatively. Clinical examination included best-corrected visual acuity (BCVA), intraocular pressure (IOP) using Goldman applanation tonometry (GAT), anterior segment examination, slit-lamp biomicroscopy using 90D, and a peripheral fundus examination using the indirect ophthalmoscope with the + 20D lens at each follow-up visit. Color fundus photographs were taken at each follow-up visit. A 5-line raster OCT scan was performed on follow-up visits to look at the remnants of ERM and recurrence of ERM, if any.

The study’s primary outcomes included the percentage of the eyes where complete ERM removal was possible without staining in groups A and B, associated intraoperative and postoperative complications, and BCVA at 3 months of follow-up. Secondary outcomes included total surgical time and ERM peel time.

Statistical analysis was done with the help of ©SPSS version 26 for Windows (IBM Inc., Chicago, IL, USA). All the measurable data was checked for normality using the Kolmogorov-Smirnov test within each group. BCVA was converted to LogMAR for statistical analysis. A p-value <0.05 was considered statistically significant.

## Results

Thirty patients diagnosed with ERM requiring surgical intervention were included in the study. There were 17 males (56.7%) and 13 females (43.3%). The mean age of the patients was 65.3 ± 10.26, ranging from 29 to 84 years. Of these 30 patients, 18 (60%) were diagnosed with idiopathic ERM, and the rest, 12 (40%), were diagnosed with secondary ERM. Of these 12 cases of secondary ERM, most were post-cataract surgery (7, i.e., 59%), 3 (25%) were post-vasculitis, and 1 case each (8%) was due to retinal vein occlusion (CRVO) and post-vitreoretinal surgery.

**Fig. 1:**
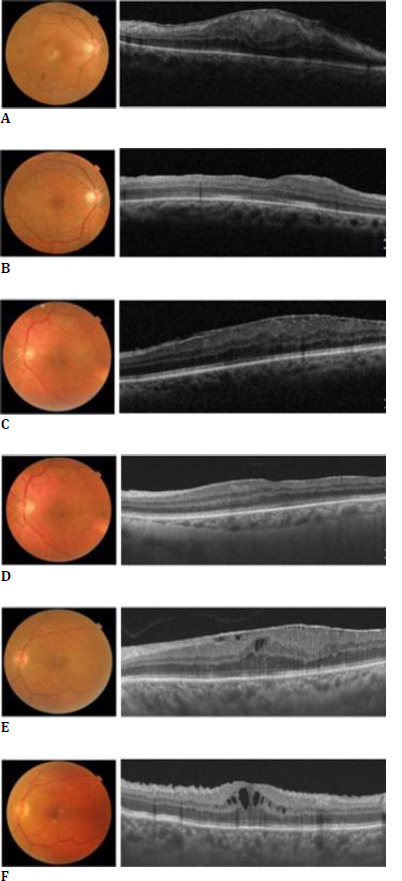
Preoperative and postoperative color fundus photograph and OCT in group A (**A, B**), group B (**C, D**), and group C (**E, F**)

Fourteen (46.6%) patients were diagnosed with stage 3 ERM, and 16 (53.3%) had stage 4 ERM. Complete ERM peeling was possible in 8 eyes (80%) from group A and seven eyes (70%) from group B. In the remaining eyes, two in group A and three in group B, BBG dye was used to identify the ERM and complete peeling. **Fig. 1** shows preoperative and postoperative images of ERM removal in groups A, B, and C.

No intraoperative complications were encountered in any of the groups. Postoperatively, one patient in group C had residual extrafoveal ERM on OCT at one week after surgery, which resulted in the recurrence of ERM in the same eye on a subsequent follow-up visit. No other complications, like increased IOP, endophthalmitis, or retinal detachment, were noted postoperatively in any of the groups.

The mean BCVA of group A was 0.75 + 0.3, group B was 0.87 + 0.4, and group C was 0.93 + 0.3 (p-value > 0.05). Postoperatively, the mean BCVA of the entire study population at 3 months improved significantly. Intragroup analysis showed a significant improvement in mean BCVA at 3 months compared to baseline (**Table 1**).

**Table 1:** Best-corrected visual acuity at baseline, 1 week, 1 month, and 3 months follow-up in all three groups

*Best-corrected visual acuity*	*Baseline*	*1 week*	*1 month*	*3 months*
**Group A**	0.75 + 0.3	1.13 ± 0.4	0.47 ± 0.2 **(p=0.703)**	0.45 ± 0.2 **(p=0.008)**
**Group B**	0.87 + 0.4	0.94 ± 0.26	0.55 ± 0.3 **(p=0.11)**	0.47 ± 0.2 **(p=0.001)**
**Group C**	0.93 + 0.3	1.02 ± 0.4	0.78 ± 0.4 **(p=0.23)**	0.49 ± 0.3 **(p=0.006)**

The mean total surgical time was 28.8 ± 8.8 minutes for group A, 24.6 ± 12.1 minutes for group B, and 40.6 ± 9.8 minutes for group C. The mean total surgical time for group A and group B was significantly lesser compared to group C (p<0.05), though the mean surgical time for group A and B was comparable (p>0.05) (**Table 2**). The mean ERM peel time was 3.2 ± 1.4 minutes for group A, 2.8 ± 1.2 minutes for group B, and 6 ± 2.4 minutes for group C. The mean ERM peel time for group A and group B was statistically lesser compared to group C (p<0.05), though the ERM peeling time for group A and B was comparable (p value>0.05) (**Table 2**).

**Table 2:** The mean total surgical time and the mean ERM peel time in all three groups (p < 0.05)

Mean total surgical time (Min)		
Group A	Group C	p value
28.8 ± 8.8	40.6 ± 9.8	0.04
Group B	Group C	0.005
24.6 ± 12.1	40.6 ± 9.8	
Group A	Group B	0.641
28.8 ± 8.8	24.6 ± 12.1	
Mean ERM peel time (Min)		
Group A	Group C	0.004
3.2 ± 1.4	6 ± 2.4	
Group B	Group C	0.001
2.8 ± 1.2	6 ± 2.4	
Group A	Group B	0.870
3.2 ± 1.4	2.8 ± 1.2	

## Discussion

MI-OCT (Group A) and 3D-HUD (Group B) ensured complete membrane peeling without any recurrence of ERM postoperatively only in 80% and 70%, respectively, without the dye, while in group C, we could achieve 100% complete removal with one recurrence. Both A and B groups had reduced total surgical and membrane peeling time compared to group C.

Though several dyes have been extensively used for ERM peeling, several clinical and experimental studies have reported potential ocular toxicities using these dyes [8-11]. As a result, the safety of these dyes for intraocular use is still debatable. Lack of standardization for the intraocular use of these dyes questions their use in clinical practice. PIONEER and DISCOVER studies have shown superior results with intraoperative OCT in visualizing real-time vitreoretinal architectural details and detecting residual membranes, which are otherwise impossible with en face visualization of tissue using SOM [12,13].

The study’s primary outcome was to assess the completeness of membrane removal in all three groups. Two patients in group A and three in group B required dye for intraoperative identification of the ERM. Minimal concentration and a very short exposure time (<30 sec) of dye were used in these cases to enable the surgeon to initiate and lift the edge of ERM. Contrary to our study, Falkner-Radler et al. achieved peeling without staining using i-OCT in 31% of cases [4]. Dye was used in the rest of the cases either due to the presence of flat ERMs or the inability to elevate the edge of ERM in the study by Falkner-Radler et al. The difference can be attributed to the fact that our study population predominantly had stage 3 and stage 4 ERM, which tended to be thicker and more adherent, and peeling initiation was easier than flat ERM. In their study on ERM peeling using i-OCT, Leisser et al. showed the need to stain in 1/3^rd^ of cases [5]. They needed to stain in cases with adherent ERM or incompletely peeled ERM. This slight difference could be attributed to the large sample size compared to ours.

No residual ERM in group A or group B highlights an added advantage of both Mi-OCT and digital filters available with 3D-HUD for intraoperative visualization of ERMs. Intraoperative OCT provides real-time images of vitreoretinal interface and tissue manipulations, thus ensuring the detection of even delicate membranes likely to be missed otherwise despite staining. Both PIONEER and DISCOVER studies showed the role of iOCT in altering surgical decision-making. iOCT could detect residual membranes in 13% of cases where the operating surgeon felt that membrane removal was complete [12,13]. 3D-HUD, coupled with digital filters, enables the surgeon to have a high-definition magnified view with an enhanced depth of focus. This allows the surgeon to visualize minor surgical details, enabling a complete membrane removal.

Recurrence of ERM was observed in only one patient (10%) in group C as opposed to no recurrence in groups A and B. Our results were consistent with the recurrence rate reported by previous studies, which have shown recurrence rates of approximately 5-12% where additional ILM peeling was not done [14,15]. Recurrent ERMs are usually mild and require re-surgery in approximately 2% of cases [14]. The recurrence of ERM postoperatively is attributed primarily to residual patches of ERM left over, which are sometimes challenging to detect intraoperatively, and the same was the case in our study. Studies have shown the effectiveness of ILM peeling and ERM peeling in preventing the recurrence of ERM [16-18]. Peeling ILM ensures complete removal of residual epiretinal tissue and removes any scaffold over which residual membranes can grow. But, ILM peeling is a traumatic surgical procedure associated with various microstructural complications [19-21]. We believe ensuring a complete ERM removal is a better alternative than peeling ILM and causing additional iatrogenic trauma to retinal architecture.

Though the improvement in BCVA at 1-month was insignificant, BCVA at 3 months was significantly better than baseline in all three groups. Our results were consistent with the ones of Ehlers et al., where patients had a significant improvement functionally and anatomically at 3 months of follow-up [22]. Even if we had a limited follow-up period of 3 months, studies have shown both anatomical and functional outcomes to improve over 6-12 months following ERM peeling. The intergroup analysis of change in BCVA at 3 months from baseline was statistically insignificant, highlighting that all three surgical techniques were equally effective in functional outcomes.

Our study’s total surgical time and ERM peel time were statistically lesser with Mi-OCT and 3D-HUD than with conventional SOM. This is, in contrast, to a survey by Talcott et al., which showed that even though total surgical time was similar, membrane peeling was significantly longer with a 3D-HUD group than with conventional SOM [23]. This difference can primarily be attributed to the learning curve associated with using 3D-HUD. Contrary to our study, where all surgeries were performed by a single surgeon with more than 18 years of surgical experience, surgeries in their study were performed by multiple surgeons, including vitreo-retinal fellows, who had different surgical expertise, thus subjectively decreasing the ease of use. Secondly, staining with dye is an additional surgical maneuver that adds to the total surgical time. In most cases, we did not use dye in groups A and B, thus reducing surgical time.

Additionally, DISCOVER and PIONEER studies have shown the advantage of i-OCT in eliminating unnecessary surgical maneuvers [12,13]. Without i-OCT, we believe surgeons will tend to perform additional surgical maneuvers, such as restaining with dye and unnecessarily peeling in doubtful areas. This not only causes iatrogenic damage and dye-related toxicity to the retina but also adds to the total surgical time.

Our pilot study highlighted that newer technologies, Mi-OCT and 3D-HUD, had no added advantage over SOM at three months follow-up. Moreover, it had a small sample size and a short follow-up duration. Secondly, we did not study any side effects related to the use of dye in group C and did not compare it with other groups. Similar studies with a longer follow-up can address the above issues. Lastly, the high cost of Mi-OCT and 3D-HUD makes it difficult for every surgeon to include them in his surgical armamentarium.

## Conclusion

To conclude, newer advancements like Mi-OCT and 3D-HUD hold a promising future in ophthalmic surgeries, especially macular surgeries. However, these devices are non-superior to SOM and do not have any additional advantage in structural and functional outcomes for ERM surgery at three months’ follow-up.

## Presentation in an event

The manuscript was submitted as an e-poster in the 14th APVRS CONGRESS (Dec. 11- 12, 2021).
